# Research advances in key genes and regulatory mechanisms of posttranslational modifications in Parkinson’s disease

**DOI:** 10.1371/journal.pone.0354097

**Published:** 2026-07-24

**Authors:** Bangzhi Wang, Zhuo Huang, Rong Wang, Chaolin Zhu, Shijiang Ma, Minghong Wang

**Affiliations:** 1 Yunnan University of Chinese Medicine, Kunming, Yunnan, China; 2 Yunnan Provincial Hospital of Chinese Medicine, Kunming, Yunnan, China; Huazhong Agriculture University, CHINA

## Abstract

**Background:**

The genesis of Parkinson’s disease (PD), a common central neurodegenerative disorder, involves dysregulation of protein posttranslational modifications (PTM). The primary objective of this study was to screen key PTM-associated genes (PTMGs) serving as diagnostic indicators and potential therapeutic targets in PD.

**Methods:**

Peripheral blood transcriptomic data for PD cohorts and healthy controls were retrieved from publicly accessible repositories. Candidate genes were identified by overlapping differentially expressed genes (DEGs) with established PTMGs via differential expression profiling. Machine learning-based screening approaches were used for the selection of feature genes. Key genes were validated via receiver operating characteristic curve assessment combined with verification of expression levels. Subsequently, enrichment analysis, immune infiltration assessment, chromosome mapping, prediction of ribonucleic acid (RNA) modification sites, and compound screening were further explored.

**Results:**

A total of 404 DEGs were identified, 19 of which overalpped with PTMGs and were thus selected as candidate genes. ML-based analysis narrowed these to eight feature genes, among which those coding for beta-1,4-galactosyltransferase 3 (*B4GALT3*), ring finger and FYVE-like domain-containing E3 ubiquitin protein ligase (*RFFL*), and GABA type A receptor-associated protein (*GABARAP*) were validated as key genes based on their diagnostic performance and consistent downregulation in the PD group (*p* < 0.05). Gene set enrichment analysis demonstrated significant enrichment within immune signaling cascades. Analysis of immune cell infiltration revealed diminished populations of activated B lymphocytes, activated CD4-positive T cells, and natural killer T cell subsets in the PD group, which exhibited predominantly positive associations with the identified key genes (*p* < 0.05). Chromosome mapping localized *B4GALT3* to chromosome 1 and *RFFL*/*GABARAP* to chromosome 17. High-confidence m^6^A methylation sites were predicted for *B4GALT3* and *RFFL*. Compound screening identified 34 potential compounds targeting these genes, including valproic acid and phenobarbital.

**Conclusion:**

This study identified *B4GALT3*, *RFFL*, and *GABARAP* as key PTMGs in PD, highlighting their roles in PD genesis and potential as diagnostic biomarkers.

## 1. Introduction

Parkinson’s disease (PD) is a prevalent neurodegenerative condition affecting the central nervous system. Its hallmark pathological feature involves aberrant accumulation of α-Syn within Lewy bodies localized to the nigrostriatal pathway. This pathological process triggers substantial depletion of dopamine within the substantia nigra pars compacta. Consequently, affected individuals manifest a spectrum of motor disturbances, including slowed movement execution, abnormalities in posture and ambulation, and tremor at rest. Additionally, non-motor manifestations also occur, including disrupted sleep architecture, dysregulation of autonomic nervous system function, and deficits spanning the psychiatric and cognitive domains [1,2]. Approximately 1.2% of people aged 65 years and older are affected by PD, with 5%–10% of cases attributable to familial genetic mutations [2]. In recent decades, about 100 different genes or loci have been found to be associated with susceptibility to PD [3]. However, the molecular mechanisms underlying this disease are still not fully understood [3], which not only implies a knowledge gap with regard to the genetics of PD, but is also the reason for the current lack of precise and effective molecular targets for PD diagnosis and treatment [4].

Evidence from alternative prevalent neurodegenerative conditions has established that dysregulated posttranslational modification (PTM) of proteins plays a critical role in disease progression, thereby offering valuable insights for investigating the molecular underpinnings of PD. In Alzheimer’s disease, pathological entities including neurofibrillary tangles, senile plaques, and amyloid β fragments demonstrate strong linkage to declining memory function, compromised cognitive performance, attenuated synaptic plasticity, and accelerated disease trajectory [5]. PTM adds various chemical fractions to the protein of interest, including phosphates, acyls, methyl groups, and glycan groups, catalyzed by specific enzymes; a process that not only affects the structural integrity of proteins, but also often causes them to lose or enhance their normal function [6]. Abnormal PTM of various proteins, such as amyloid precursor protein, secretases, various kinases, and phosphatases, has been implicated in the development of neurodegenerative diseases [7]. PTM governs protein functionality, expression levels, and intermolecular interactions by modulating protein characteristics and stability profiles [8]. This modification process influences the hydrophobic properties of proteins while triggering structural and conformational alterations, consequently determining protein functional capacities and protein–protein interaction dynamics [5]. However, prospective biomarkers and underlying regulatory networks remain incompletely characterized. Consequently, investigating PTM aberrations and deciphering their molecular underpinnings may establish the foundations for rational and efficacious therapeutic strategies targeting neurodegenerative conditions [8]. PTM is likely to be involved in PD pathology by regulating α-synuclein folding and aggregation, which also suggests that PTM-related molecules can be potential candidates for PD biomarkers.

Advances in bioinformatics and genomic technologies have greatly advanced PD research, particularly through the integration of large-scale transcriptomic datasets from public repositories such as the Gene Expression Omnibus (GEO) database. These datasets enable systematic analysis of gene expression profiles in patients with PD versus healthy controls, facilitating the identification of candidate genes and biological pathways associated with PD progression [9]. However, the molecular mechanisms underlying PD remain incompletely elucidated, hindering the discovery of precise early diagnostic biomarkers and therapeutic targets. Therefore, exploring novel biomarkers of PD is critical for early detection and intervention.

Machine learning (ML) algorithms offer robust tools for analyzing potential associations in high-dimensional data. The Least Absolute Shrinkage and Selection Operator (LASSO) addresses dimensionality reduction challenges in high-dimensional genetic data through variable selection, whereas the eXtreme Gradient Boosting (XGBoost) algorithm optimizes the performance of integrated models via iteration. The combination of these algorithms has been successfully applied to identify PD biomarkers [9], enabling precise screening of feature genes closely linked to PD genesis.

Building on this previous research, the present study integrated peripheral blood transcriptomic data for patients with PD and healthy controls obtained from the GEO database (GSE18838 as the training set and GSE22491 as the validation set). First, differentially expressed genes (DEGs) related to PD were screened via differential expression analysis and intersected with known PTM-related genes (PTMGs) to obtain candidate genes. Subsequently, the LASSO and XGBoost ML algorithms were used to filter feature genes associated with PTM function. Core biomarkers were determined by evaluating diagnostic efficacy via receiver operating characteristic (ROC) curve analysis and verification of expression levels. To comprehensively characterize the regulatory networks governing these biomarkers, multiple analytical approaches were adopted, including chromosomal mapping, RNA m^6^A methylation site prediction, immune infiltration profiling (elucidating biomarker-immune cell associations), and compound screening. Overall, this investigation elucidates the functional contributions of key PTMGs to PD pathophysiology, thereby identifying prospective diagnostic biomarkers for clinical PD assessment alongside novel therapeutic intervention targets.

## 2. Materials and methods

### 2.1. Data sources

The gene expression profile data for the training set (GSE18838) and validation set (GSE22491) were downloaded from the GEO database (https://www.ncbi.nlm.nih.gov/geo; accessed on May 21, 2025). The training dataset contained peripheral blood transcriptomic data for 17 patients with PD and 11 normal controls and was generated on the GPL5175 platform using chip sequencing technology. The validation dataset encompassed transcriptomic profiles derived from peripheral blood mononuclear cells obtained from 10 patients with PD and 8 healthy controls and was generated via chip sequencing methodology on the GPL6480 platform. Furthermore, from the references, 822 unique PTMGs were extracted (**S1 Table**) [10] (accessed on May 21, /2025).

### 2.2. Differential expression analysis

DEGs between the PD and control groups in the GSE18838 training set were determined using the R package “limma” (v 3.54.0) [11] [absolute log_2_-fold change (|log_2_ Fold Change (FC)|) > 0.5, *p* < 0.05, Benjamini–Hochberg multiple test correction]. The R package “ggplot2” (v 3.5.1) [12] was used to generate a volcano map, while the “ComplexHeatmap” package (v 2.14.0) [13] was used to construct a hierarchical clustering heatmap to show the top 20 DEGs (the 10 most significant upregulated and downregulated genes selected based on |log_2_FC| values in descending order).

### 2.3. Identification of candidated genes

To identify the candidate PTMGs in PD, the DEGs obtained from analysis of the training set (GSE18838) were intersected with the 822 extracted PTMGs, and the overlapping genes were defined as candidate genes. The R package “ggvenn” (v 0.1.9) [14] was used to visualize the intersection between DEGs and PTMGs.

### 2.4. Gene Ontology and Kyoto Encyclopedia of Genes and Genomes enrichment analyses

To investigate biological functionalities associated with candidate genes, Gene Ontology (GO) enrichment analysis for biological processes (BPs), cellular components (CCs), and molecular functions (MFs) (*p* < 0.05) was conducted via the R package “clusterProfiler” (v 4.6.2) [15]. Within each domain, enrichment outcomes were ranked from high to low based on gene count, followed by extraction of the five most statistically significant terms for graphical representation. To characterize in more detail crucial signaling cascades involving candidate genes, Kyoto Encyclopedia of Genes and Genomes (KEGG) pathway enrichment analysis was conducted (*p* < 0.05) using the R package “clusterProfiler” (v 4.6.2). Statistically significant KEGG pathways were ranked from high to low based on gene enrichment frequency, with the 10 most prominent pathways subsequently selected for visualization.

### 2.5. Protein–Protein Interaction analysis

The search tool for the retrieval of interacting genes/proteins (STRING) database (https://string-db.org) provides a comprehensive resource of information on protein–protein interactions and assigns a composite interaction score to predicted or experimentally validated interactions. To explore associations between candidate genes at the protein level, the list of candidate genes was uploaded to the STRING database for protein–protein interaction (PPI) network analysis (composite interaction score ≥ 0.15). The results obtained were exported and visualized in Cytoscape (v 3.7.2) [16].

### 2.6. Machine learning algorithms

To ensure robust feature gene selection from candidate genes, we employed two distinct ML algorithms, LASSO and XGBoost. The rationale for selecting these algorithms is rooted in their complementary strengths in handling high-dimensional transcriptomic data, particularly in small-sample settings such as the present study (n = 28 in the training set [17].

The LASSO algorithm is a biased estimation approach that achieves variable screening and multicollinearity resolution through incorporation of L1 regularization penalty components. It compresses some regression coefficients by constructing a penalty function while setting other coefficients to zero, resulting in a more refined model. Candidate genes in the training dataset (GSE18838) were analyzed using the R package “glmnet” (v 4.1−4) [18]. The random seed was set to 25 to ensure reproducibility. Fivefold cross-validation was employed to determine the optimal regularization parameter λ (lambda), which was defined as the value yielding minimal partial likelihood deviance within the model framework. Under this optimal λ value, genes with non-zero coefficients, which are the characteristic genes screened by the LASSO algorithm, are screened out.

The XGBoost algorithm iteratively builds multiple weak evaluators (decision trees) and combines them into a single strong evaluator. With each iteration, the newly constructed tree is designed to correct for the residuals of the pre-order model assembly, thereby gradually improving the overall prediction performance of the model. Here, the R package “xgboost” (v 1.7.3.1) [19] was used to build an XGBoost model for the GSE18838 training set. Key parameters were set as follows: maximum depth of the tree (max_depth) = 6; learning rate (eta) = 0.3; objective function, binary:logistic; and number of iterations (nround) = 200. Model stability was evaluated by fixing the random seed via set.seed(1) to ensure the reproducibility of a single run. During model training, the subset of features used when the model achieved the strongest evaluation ability (i.e., optimal performance) was determined by monitoring the performance of the model on the validation set ([*please rephrase this part]* such as error or specifying evaluation indicators), and the corresponding feature genes were identified as the feature genes screened by the XGBoost algorithm. The feature genes screened out by the analyses via LASSO and XGBoost were then intersected, and the overlap between them was visualized by using the R package “ggvenn” (v 0.1.9). The intersecting genes were finally selected for subsequent in-depth analysis.

### 2.7. ROC analysis

To assess the diagnostic ability of feature genes to distinguish between the PD from control groups, the ROC was analyzed in the training (GSE18838) and validation (GSE22491) sets. The area under the curve (AUC) for the ROC curve was calculated using the R package “pROC” [20] based on the profiles of feature genes. Genes with an AUC value of >0.7 were defined as candidate key genes.

### 2.8. Gene expression analysis

To corroborate alterations in the expression of candidate key genes under PD conditions, the training (GSE18838) and validation (GSE22491) sets were separately subjected to expression profiling. The Wilcoxon test enabled comparative assessment of expression magnitudes for individual candidate key genes across PD cohorts versus control cohorts (*p* < 0.05). The expression patterns of candidate genes for both PD and control cohorts were visualized using a boxplot generated via the R package “ggplot2” (v 3.5.1). Candidate key genes were identified as those demonstrating statistically significant differential expression (*p* < 0.05) alongside concordant directionality in expression (upregulation or downregulation) when comparing PD cohorts against control cohorts across both the training (GSE18838) and validation (GSE22491) datasets.

### 2.9. Gene set enrichment analysis

Gene set enrichment analysis (GSEA) identifies phenotype-related functional pathways by detecting the distribution characteristics of predefined gene sets in a list of sequenced genes and captures subtle changes in the centralized synergistic expression of genes. The Spearman correlation coefficient (cor) for key genes and genome-wide expression data in the training set (GSE18838) was calculated using the R package “psych” (v 2.2.9) [21]^,^ and all genes were sorted from highest to lowest. The predefined reference gene sets used were the default gene sets from the Molecular Signatures Database (MSigDB) (https://www.gsea-msigdb.org/gsea/msigdb). The gene list was sorted using the R package “clusterProfiler” (v 4.6.2). The following significance thresholds were applied for enrichment outcomes: *p* < 0.05; absolute normalized enrichment score (|NES|) > 1; and false discovery rate (FDR) < 0.25. Subsequently, the five pathways exhibiting minimal p-values within GSEA outcomes were visualized using the R package “enrichplot” (v 1.18.0) [22].

### 2.10 Analysis of immunoinfiltration

Two complementary algorithms were employed to comprehensively evaluate the differences in immune cell infiltration between the PD and control groups. First, we performed single-sample Gene Set Enrichment Analysis (ssGSEA) using the R package GSVA (v 1.46.0) [23] to quantify the sample-specific abundance of 28 infiltrating immune cell subtypes in the training set (GSE18838). The parameters for analysis were strictly set as follows: method = “ssgsea,” kcdf = “Gaussian,” and abs.ranking = TRUE. This enabled the calculation of standardized enrichment scores based on the rank of gene expression. The signature gene sets corresponding to the 28 immune cell subtypes were directly obtained from a previously published study [24]. The original expression matrix downloaded from the GEO database was directly used for analysis. To further expand the dimension for detecting the immune cell landscape and validate the robustness of the ssGSEA results, we simultaneously applied the xCell algorithm using the R package IOBR (v 0.99.9) [25] to calculate the enrichment scores for 64 immune and stromal cell types in the same training set, thereby providing a more detailed resolution of the PD immune microenvironment at the subpopulation level. We then applied the Wilcoxon rank‑sum test to the immune cell infiltration scores derived from both ssGSEA and xCell to compare the differences in infiltration levels between the PD group and the healthy control group. To avoid type I error inflation due to multiple hypothesis testing, we uniformly applied the Benjamini–Hochberg method to control the FDR, with an adjusted *p* < 0.05 as the statistical threshold for identifying immune cells with different levels of infiltration. Subsequently, we selected the consistently differential immune cell subsets identified by algorithms and used Spearman’s rank correlation analysis to evaluate the strength of the association between their infiltration scores and the expression levels of hub genes, with the screening criteria set as |cor| > 0.3 and p < 0.05. Finally, we visualized the correlation heatmaps using the R packages “pheatmap” (v 1.0.12) [26] and “corrplot” (v 0.92) [27] to intuitively display the regulatory network linking key genes and immune cells.

### 2.11. Chromosomal localization of key genes

To localize key genes on the chromosomes, [*please rephrase after this point*] biomarker distribution across chromosomes was visualized via the R package “Circos” (v 1.2.2) [28].

### 2.12. Analysis of modification sites

The sequence-based RNA adenosine methylation site predictor (SRAMP) (http://www.cuilab.cn/sramp) was employed to predict m^6^A modification sites within key genes. The “Full Profile” model was adopted, which integrates nucleotide sequence attributes alongside evolutionary conservation data to make comprehensive predictions. The default “high-confidence” threshold was maintained. [*Please rephrase this sentence]* RNA Secondary Structure Feature Check “Include RNA structure feature” (requires RNAfold support). Loci with a probability value > 0.6 were retained as high-confidence candidates.

### 2.13. Compound prediction

The Drug Signatures Database (DSigDB) (https://dsigdb.tanlab.org/DSigDBv1.0) was used to explore compounds targeting key genes by examining the interactions between key genes and known compounds. Compound–gene interaction networks were built in Cytoscape (v 3.7.2).

### 2.14. Statistical analysis

Bioinformatics analysis was performed in R (v 4.2.2). Inter-group data comparisons were conducted using the Wilcoxon test. Statistical significance was set at *p* < 0.05.

## 3. Results

### 3.1. Identification and functional analysis of candidate genes

**Fig 1:**
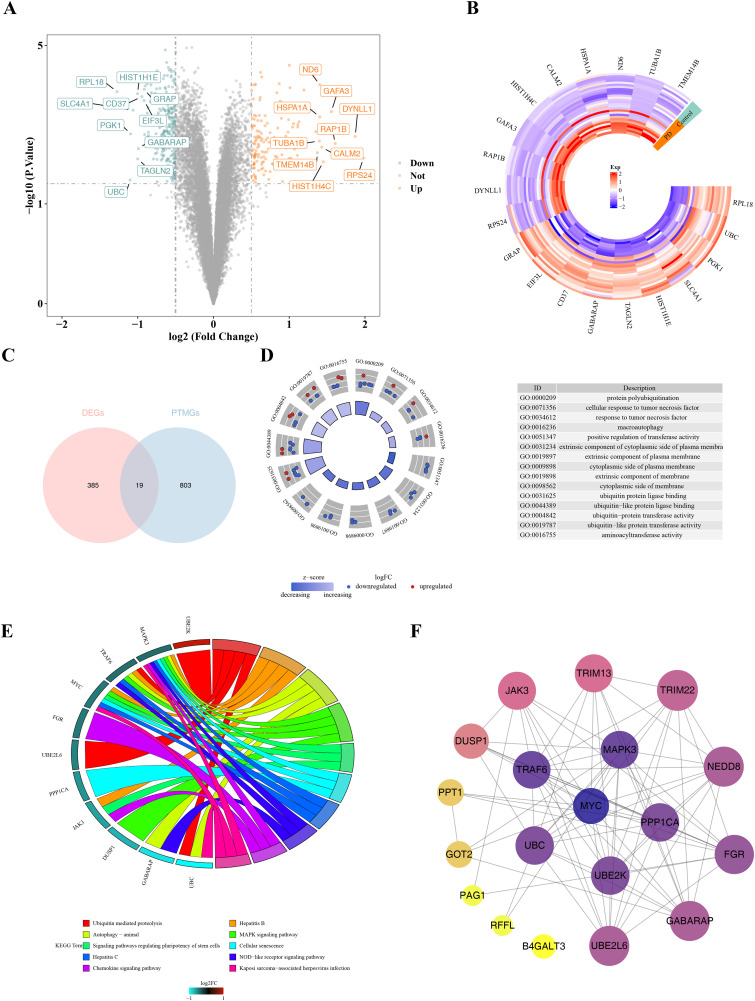
Identification of candidate genes. (**A**) Volcano plot of differentially expressed genes (DEGs). Yellow and green dots represent upregulated and downregulated genes, respectively, while gray dots indicate genes showing no significant differential expression. (**B**) Heatmap of DEGs showing the expression of the top 10 upregulated and top 10 downregulated genes across samples. The red and blue colors indicate high and low expression, respectively. (**C**) Venn diagram of candidate genes. The pink and green colors indicate DEGs and protein posttranslational modification-related genes (PTMGs), while the overlapping area represents candidate genes. (**D**) Circular “Bagua” plot for the results of GO enrichment analysis of candidate genes. (**E**) Chord diagram for the results of KEGG enrichment analysis of candidate genes. (**F**) STRING network analysis of candidate genes. [*Please rephrase after this point*] Colors reflect the degree of each candidate gene. Higher degree values are indicated by a gradient shift from yellow to purple.

Analysis of differential expression between PD and control groups yielded 404 DEGs (|log_2_FC| > 0.5, *p* < 0.05). Among them, 145 were upregulated and 259 were downregulated in PD cohorts (Fig 1A-B). To explore the role of PTMs in PD genesis, these 404 DEGs were intersected with 822 known PTMGs. A total of 19 overlapping genes were obtained, which were defined as candidate genes (**Fig 1C**). GO analysis revealed significant enrichment of these genes across 413 BPs, 18 CCs, and 61 MFs. Among the significantly enriched BPs were protein polyubiquitination, cellular response to tumor necrosis factor, macroautophagy, and positive regulation of transferase activity activity. Significantly enriched CCs incuded the extrinsic component of the cytoplasmic side of the plasma membrane and the extrinsic component of the plasma membrane. For MFs, significantly enriched terms predominantly included ubiquitin protein ligase binding, ubiquitin -like protein transferase activity, and aminoacyltransferase activity (**Fig 1D**, **S2 Table**). KEGG pathway analysis additionally demonstrated substantial enrichment of candidate genes within 77 signaling cascades, encompassing ubiquitin-mediated proteolysis, hepatitis B, autophagy-animal, MAPK signaling, pathways regulating stem cell pluripotency, and NOD-like receptor signaling (**Fig 1E**, **S3 Table**). PPI network analysis showed that the 19 candidate genes formed a complex network of 81 interaction pairs at the protein level, with *MYC*, *TRAF6*, and *MAPK3* exhibiting extensive interactions with proteins encoded by multiple genes (**Fig 1F**).

### 3.2. Identification of eight feature genes via ML

**Fig 2:**
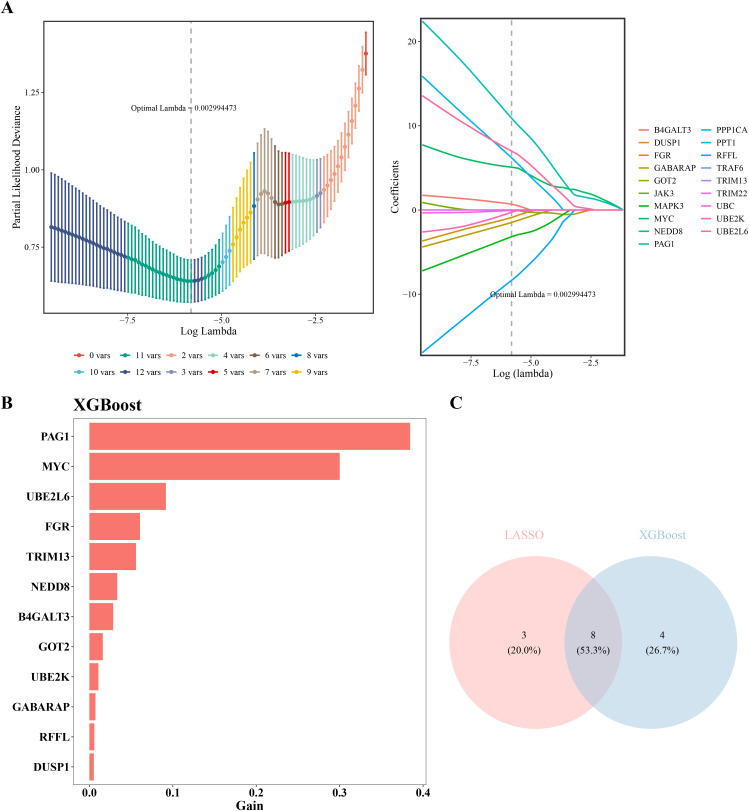
Machine learning was used to screen the datasets for feature genes. (**A**) Graphs showing the LASSO logical coefficient penalty values and the cross-validation error curve. (**B**) Ranking of the relative importance of explanatory variables in the model. (**C**) Venn diagram showing the selection of feature genes.

To precisely pinpoint genes closely associated with the pathological mechanisms underlying PD, two ML algorithmic approaches were employed for screening 19 candidate genes. Regression modeling using LASSO demonstrated optimal performance at the minimal penalty coefficient λ value (lambda = 0.002994473), yielding 11 prospective signature genes: ubiquitin conjugating enzyme E2 K (*UBE2K*), phosphoprotein membrane anchor with glycosphingolipid microdomains 1 (*PAG1*), MYC proto-oncogene (*MYC*), mitogen-activated protein kinase 3 (*MAPK3*), palmitoyl protein thioesterase1 (*PPT1*), ubiquitin conjugating enzyme E2 L6 (*UBE2L6*), fetal growth restriction (*FGR*), ubiquitin C (*UBC*), *B4GALT3*, *RFFL*, and *GABARAP* (Fig 2A). The XGBoost algorithm, which was used to evaluate the importance of candidate genes, revealed that the model performed best when selecting the top 12 feature genes, i.e., *PAG1*, *MYC*, *UBE2L6*, *FGR*, *TRIM13*, *NEDD8*, *B4GALT3*, *GOT2*, *UBE2K*, *GABARAP*, *RFFL*, and *DUSP1* (Fig 2B). The results obtained using the two algorithms were intersected, and eight feature genes were finally identified: *UBE2K*, *PAG1*, *MYC*, *UBE2L6*, *FGR*, *B4GALT3*, *RFFL*, and *GABARAP* (Fig 2C).

### 3.3. Diagnostic efficacy and expression patterns of feature genes

**Fig 3:**
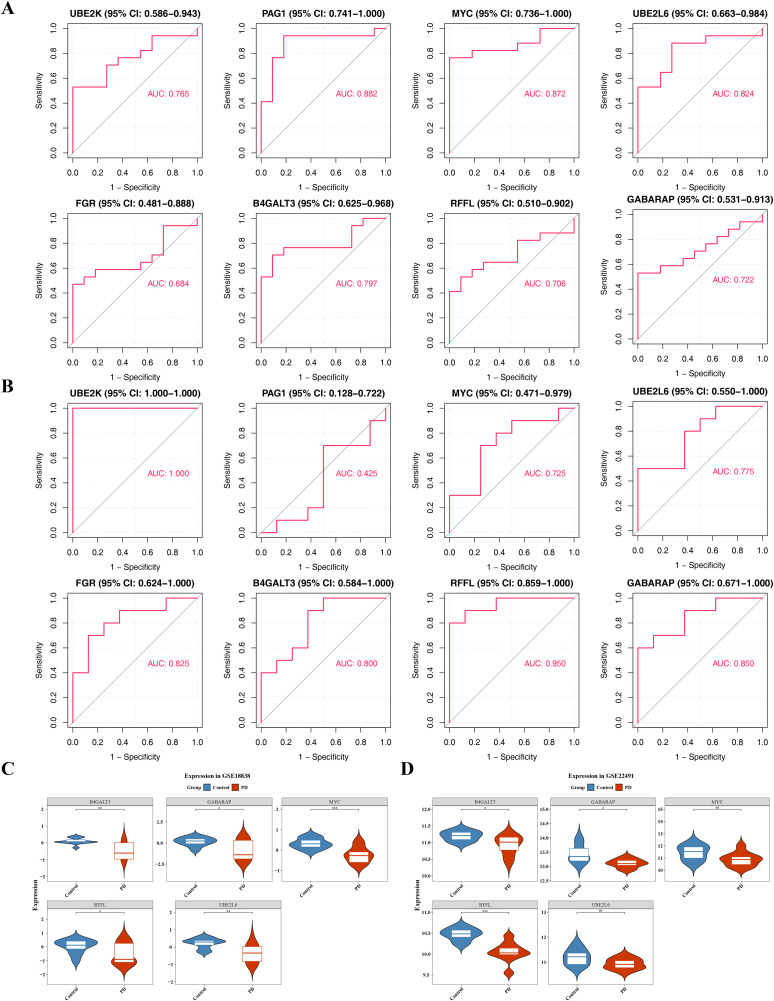
Identification of key genes. (**A**) ROC curve of feature genes in the training set. (**B**) ROC curve of feature genes in the validation set. (**C**) Differences in the expression levels of feature genes in the training set. (**D**) Differences in the expression levels of feature genes in the validation set. Significance levels: *, *p* < 0.05; **, *p* < 0.01; and ***, *p* < 0.001.

To evaluate the diagnostic potential of the eight identified feature genes (*UBE2K*, *PAG1*, *MYC*, *UBE2L6*, *FGR*, *B4GALT3*, *RFFL*, and *GABARAP*), ROC curves were analyzed in both the training and validation sets. In the training set, the AUC values for *UBE2K*, *PAG1*, *MYC*, *UBE2L6*, *B4GALT3*, *RFFL*, and *GABARAP* were all > 0.7, indicating that the genes had significant diagnostic efficacy, whereas the AUC value of *FGR* was below this threshold (**Fig 3A**). In the validation set, only *MYC*, *UBE2L6*, *B4GALT3*, *RFFL*, and *GABARAP* had AUC values >0.7, indicating stable diagnostic efficacy (**Fig 3B**). Therefore, these five were identified as candidate key genes. Gene expression analysis showed that their expression levels were significantly lower in the PD group than in the control group (*p* < 0.05) (**Fig 3C**). In the validation set, the expression levels of *B4GALT3*, *GABARAP*, and *RFFL* in the PD group were still significantly downregulated (*p* < 0.05) (**Fig 3D**). However, no significant differences in expression were observed between *MYC* and *UBE2L6*, which may be attributed to the reduced reproducibility of gene expression signals due to discrepancies in sample sources, processing procedures, and detection platforms between the validation and training sets. Overall, the selection of *B4GALT3*, *GABARAP* and *RFFL* as key genes based on dual validation of their diagnostic efficacy and expression patterns provides a new strategy for the early detection of PD.

### 3.4. GSEA-based pathway enrichment analysis of key genes

**Fig 4:**
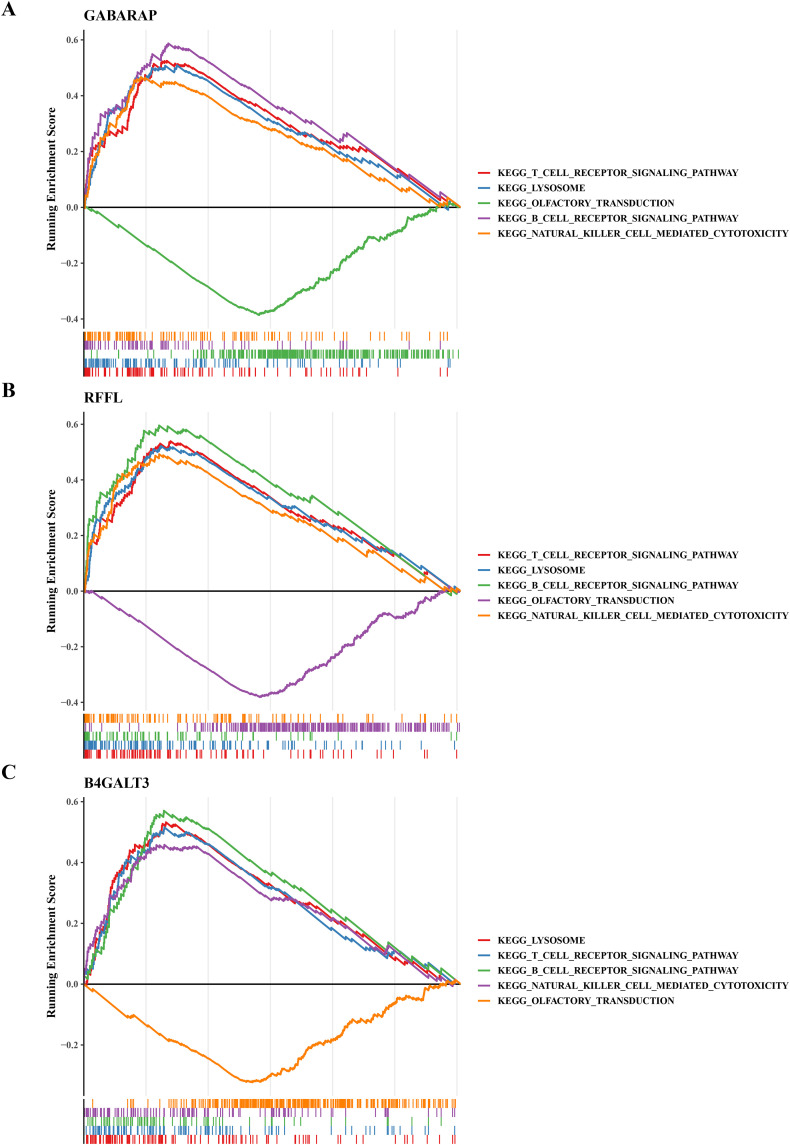
Gene set enrichment analysis (GSEA) of key genes: (A) *B4GALT3*, (B) *GABARAP*, and (C) *RFFL.*

To elucidate the functional mechanism of the three selected key genes (*B4GALT3*, *GABARAP*, and *RFFL*) in PD, their regulatory pathways were examined via GSEA. The results showed that *GABARAP*, *RFFL*, *B4GALT3* were significantly enriched in 89, 97, and 83 pathways, respectively (*p* < 0.05, |NES| > 1, FDR < 0.25), with all three genes being specifically enriched in T cell receptor signaling, lysosomal function (lysosome pathway), and olfactory transduction (olfactory pathway) (Fig 4A-C, S4-S6 Table). The GSEA results for these key genes revealed that they are involved in PD pathology through multiple dimensions.

### 3.5. Characteristics of immune cell infiltration and association with key genes

**Fig 5:**
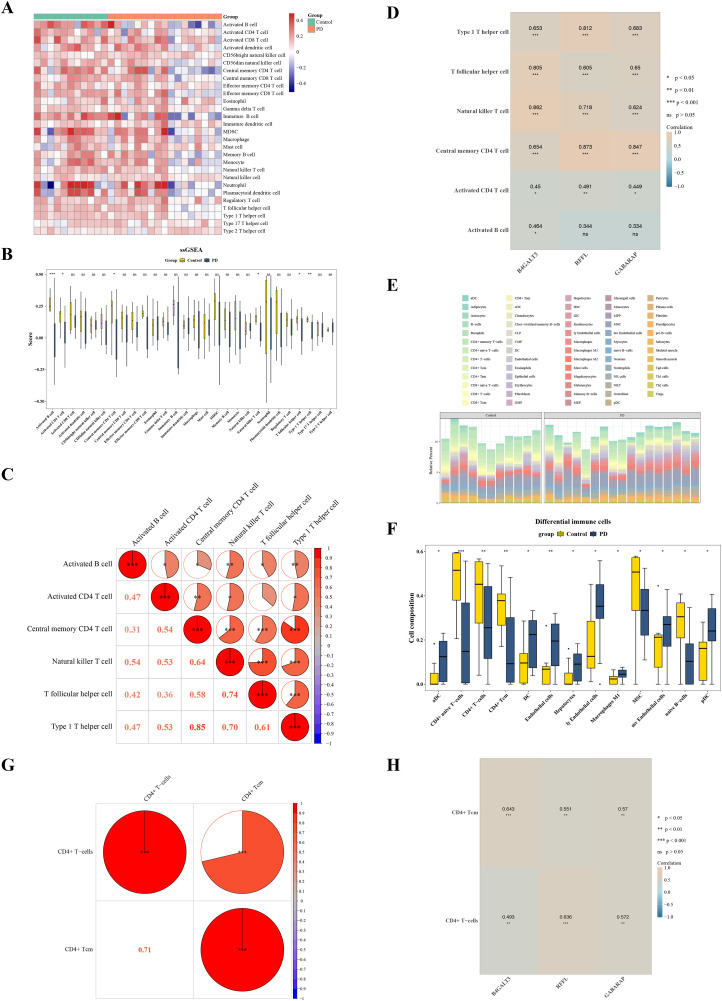
Immune infiltration analysis. (**A**) Heatmap of 28 types of infiltrating immune cells in the PD and control groups. (**B**) Differences in immune cell types between the PD and control groups. (**C**) Heatmap showing the correlations between different immune cell types. (**D**) Heatmap showing the correlations between key genes and different immune cells. **(E-F)** Heatmap and box plot of different immune cell types based on the xCell algorithm. **(G)** Correlation heatmap of different immune cell types based on the xCell algorithm. **(H)** Heatmap showing the correlation between key genes and immune cell types identified by the xCell algorithm. Significance levels: ns, no significant difference; *, *p* < 0.05; **, *p* < 0.01; ***, p < 0.001.

Immune infiltration profiling revealed substantially lower infiltration magnitudes across six immune cell populations in the PD group than in the control (*p* < 0.05). These populations included activated B cells, activated CD4 T cells, central memory CD4 T (CD4 Tcm) cells, natural killer T cells, T follicular helper cells, and Type 1 T helper cells (Fig 5A-B). Correlation analysis of immune cell populations with different infiltration levels demonstrated significant positive associations across all cell types (cor > 0.3, *p* < 0.05), with the exception of T follicular helper cells versus activated CD4 T cells. Notably, CD4 Tcm cells displayed robust positive correlation with T helper cells (cor = 0.85, *p* < 0.001). Additionally, T helper cells exhibited pronounced positive correlations with natural killer T cells (cor = 0.70, *p* < 0.001) and T follicular helper cells (cor = 0.61, *p* < 0.001) (**Fig 5C**). Subsequently, correlations between the three selected key genes (*B4GALT3*, *RFFL*, and *GABARAP*) and immune cells were analyzed to explore the role of these genes in immune regulation. The results showed a significant positive correlation between *B4GALT3* and all six immune cell types (*p* < 0.001), with the strongest association observed with natural killer T cells (cor = 0.862, *p* < 0.001) and T follicular helper cells (cor = 0.805, *p* < 0.001). *RFFL* was significantly positively correlated with CD4 Tcm cells (cor = 0.873, *p* < 0.001), T helper cells (cor = 0.812, *p* < 0.001), and natural killer T cells (cor = 0.718, *p* < 0.001). *GABARAP* exhibited a strong positive correlation with CD4 Tcm cells (cor = 0.847, *p* < 0.001). Remarkably, activated B cells demonstrated a significant positive correlation exclusively with *B4GALT3* (cor = 0.464, *p* < 0.05) (**Fig 5D**). To further validate the above findings, 64 cell types in the training set were scored using the xCell algorithm. The results showed that a total of 13 cell types differed significantly between the PD and control groups (Fig 5E-F). Notably, the CD4^+^ T cells and CD4^+^ Tcm cells identified by the xCell algorithm corresponded to the significantly different activated CD4 T cells and CD4 Tcm cells identified by the ssGSEA algorithm. Further correlation analysis revealed a significant positive correlation between CD4^+^ T cells and CD4^+^ Tcm cells identified via the xCell algorithm (cor = 0.71, *p* < 0.05) (**Fig 5G**), which was consistent with the positive correlation between activated CD4 T cells and CD4 Tcm cells in the ssGSEA algorithm (cor = 0.54, *p* < 0.05), thereby validating the reliability of the results obtained from both algorithms. Subsequently, the xCell algorithm was used to analyze the correlations between the three key genes and both CD4^+^ T cells and CD4^+^ Tcm cells, revealing significantly positive correlations with both cell types (|cor| > 0.3, *p* < 0.05) (**Fig 5H**). In summary, multiple immune cell types exhibited significantly decreased infiltration levels in the PD group, especially the CD4 T cell subsets. The key genes *B4GALT3*, *RFFL*, and *GABARAP* were significantly positively correlated with these different immune cell populations, suggesting that they may play key regulatory roles in immune dysregulation in PD.

### 3.6. Regulatory mechanisms of key genes and compound prediction

**Fig 6:**
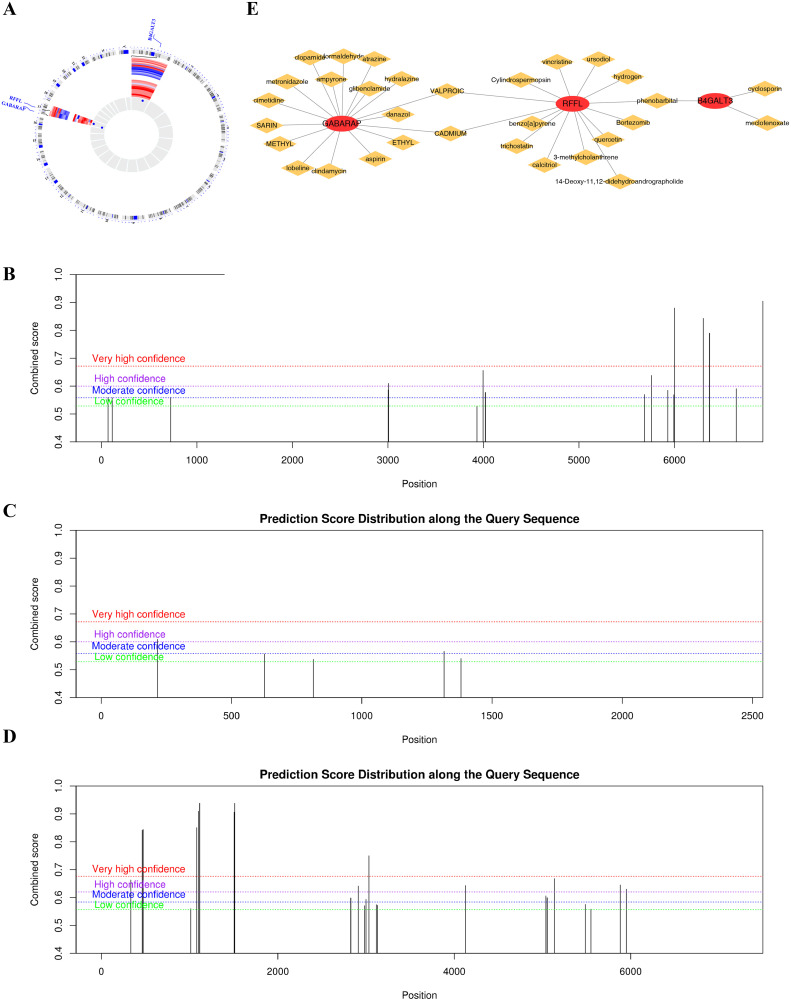
Chromosomal mapping, analysis of m^6^A modification, and compound predictions for key genes. (**A**) Chromosome localization of key genes. (**B–D**) Prediction of the m^6^A site of key genes *B4GALT3* **(B)**, *GABARAP* **(C)**, and *RFFL* **(D)**. (**E**) Prediction of compound–gene interactions for key genes. In the figure, the red and orange nodes represent mRNA (key genes) and compounds, respectively.

Chromosomal mapping, analysis of m^6^A modification, and compound predictions were carried out for *B4GALT3*, *GABARAP*, and *RFFL*. The results showed that *B4GALT3* was located on chromosome 1, whereas *GABARAP* and *RFFL* were both located on chromosome 17 (Fig 6A). Analysis of m^6^A modification revealed 16 potential m^6^A binding sites in *B4GALT3*, three of which were predicted with “very high” confidence (Fig 6B). Although five m^6^A binding sites were localized to *GABARAP*, no high-confidence modification sites were found (Fig 6C). A total of 24 m^6^A binding sites were identified in *RFFL*, eight of which with high confidence (Fig 6D). These predictions suggest that *B4GALT3* and *RFFL* may be involved in the pathological process underlying PD through m^6^A-mediated PTM, while the regulatory mechanisms of *GABARAP* may involve other epigenetic pathways, though experimental validation is required to precisely identify these processes. Compound predictions revealed that *B4GALT3*, *GABARAP*, and *RFFL* interact with 3, 17, and 14 compounds, respectively (Fig 6E). Notably, valproic acid and cadmium can affect both *GABARAP* and *RFFL*, whereas phenobarbital can act on both *RFFL* and *B4GALT3*. This multi-target regulatory model suggests that the above compounds may affect the occurrence and development of PD through synergistic regulation of key gene networks.

## 4. Discussion

PD is a complex neurodegenerative disorder whose core neurodegenerative mechanisms include genetic mutations, PTMs, and excessive levels of reactive oxygen species that drive insoluble Lewy body fibril formation and α-syn aggregation [29]. Approximately 5%–10% of PD cases are monogenic (mutations in over 20 genes), and PTMs regulate mitochondrial function, with some PD-related genes (e.g., *Parkin*, *PINK1*) encoding PTM enzymes and others (e.g., α-synuclein) undergoing PTMs to alter mitochondria [30]. Additionally, UPR/UPS/ALP mutations, epigenetic changes, and microRNA dysregulation cause protein homeostasis disruption, with deposited proteins in body fluids acting as potential early biomarkers [31]. [*Please reconsider this sentence*]This study integrated public transcriptomic data to identify three key PTMGs (*B4GALT3*, *RFFL*, *GABARAP*), alongside reduced PD immune cell infiltration, chromosome localization, m⁶A prediction, and 34 potential compounds, providing new targets for early PD diagnosis.

### 4.1. Functions and mechanisms of biomarkers

The enzyme B4GALT3, a member of the β-1,4-galactosyltransferase family, sustains immune equilibrium by modulating modifications in protein N-glycosylation. In this study, the PD group exhibited significant downregulation of *B4GALT3*. *B4GALT3*-deficient fibroblasts have been shown to secrete high levels of IL-6 and IL-8, which further activate the NF-κB pathway [32]. Relevant studies have also revealed that activated CD8 T cell infiltration is enhanced in *B4GALT3*-deficient mice, with this deficiency altering the N-glycosylation of multiple proteins, including integrin alpha L (ITGAL), which is involved in T cell activation and proliferation [33]. CD8 T cells can activate the NF-κB pathway by secreting cytokines such as IFN-γ and TNF-α. At the same time, abnormal aggregation of α-synuclein (α-syn) interacts with toll-like receptors, activating the NLRP3 inflammasome in microglia. This process leads to the release of pro-inflammatory factors via NF-κB translocation, causing mitochondrial damage and impairing dopaminergic neurons. Additionally, sustained activation of NF-κB signaling in dopaminergic neurons of the substantia nigra in PD can directly induce neuronal apoptosis [34]. Therefore, *B4GALT3* downregulation may trigger T cell overactivation via dysregulation of the glycosylation process, which in turn initiates a neuroinflammatory cascade and ultimately accelerates PD progression.

STING-induced autophagy of γ-aminobutyric acid type A receptor-associated protein (GABARAP) activates leucine-rich repeat kinase 2 (LRRK2) through the lipidation of ATG8 (a ubiquitin-like protein), and LRRK2 mutations are an important genetic factor in familial PD [35,36]. The downregulation of GABARAP observed in this study implies the involvement of this protein in *LRRK2* activation via autophagy dysfunction in PD. *GABARAP*-mediated autophagy clears misfolded proteins (e.g., α-synuclein aggregates), and reduced expression promotes the accumulation of toxic proteins. Targeting *GABARAP* may alleviate protein imbalance, preserve lysosomal homeostasis, and mitigate neuroinflammation in PD.

*RFFL* (*CARP2*) is an E3 ubiquitin ligase enzyme with localization to the endosome and plasma membrane responsible for important functions within protein degradation pathways and mitochondrial quality surveillance mechanisms [37]. Genetic alterations in the ubiquitin ligase *PRKN* (parkin RBR E3 ubiquitin protein ligase) demonstrate associations with PD genesis as well as with the impairment of mitochondrial autophagy and structure. *RFFL* interacts with PRKN (Parkin), a key protein in PD, to promote PRKN recruitment to damaged mitochondria [38]. *RFFL* also mediates lysosomal degradation of unfolded proteins such as ΔF508-CFTR via polyubiquitination. In neurons, this mechanism may be involved in the clearance of α-synuclein aggregates, and its loss of function can lead to the accumulation of toxic proteins [39]. The relationship between *RFFL* functions and PD pathology, as well as the regulatory mechanisms of B4GALT3/GABARAP collectively indicate that dysregulated protein turnover and stability contributes to PD development. These findings clarify the multidimensional mechanisms of key genes related to PD, providing novel insights into the complex processes underlying its genesis.

### 4.2. In-depth interpretation of GSEA analysis

Dysregulation of T cell receptor signalin can lead to the development of various diseases [40]. Pathogenic α-synuclein peptide-specific T cell responses can result in dopaminergic neurodegeneration, causing PD-like pathologies [41]. The enrichment of key genes in the T cell receptor signaling pathway suggests an imbalance of T cell immunoregulation in PD. Lysosomal dysfunction underlies the pathological accumulation of α-synuclein (a major component of aggregates within Lewy cells) and, among the lysosomal genes involved, *GBA1* has the greatest impact on PD risk. Lysosomal impairment has key functions in PD genesis [42]. Olfactory transduction is one of the earliest features of neurodegenerative lesions, including PD, and there are different NAD (+)-dependent deacetylase mechanisms in different regions of the brain related to olfactory pathways in different populations [43]. The present study suggests that key genes may be involved in this process by regulating olfactory receptor expression or olfactory bulb neuronal function, providing a new direction for the development of early PD biomarkers.

The B-cell receptor is a signature surface complex of B cells that regulates their activation, proliferation, and antibody secretion through Igα(CD79a)/Igβ(CD79b)-mediated signal transduction [44]. B-cell dysfunction is closely related to neuroinflammation. Therefore, the B4GALT3 of key genes in this study (regulating protein glycosylation) may affect the signaling efficiency of B-cell receptor complexes by modifying their glycosylation levels, thereby participating in the regulation of B-cell immune disorders in PD.

Natural killer (NK) cells are cytotoxic cells of the innate immune system, and myeloid-derived suppressor cells mediate immunosuppression to increase NK cytotoxicity is rarely reported [45]. NK cell-mediated cytotoxicity clears abnormal neurons and α-synuclein aggregates, the latter being a major component of Lewy bodies, and systemic depletion of NK cells has been shown to lead to neuropathological deterioration in mouse models of α-synucleinopathy. NK cells have direct and indirect regulatory functions in the context of PD [46].

### 4.3. Immune cell infiltration

Immune system dysfunction is associated with the onset and progression of PD. B-cell reduction is one of the core features of PD-related immune disorders. Within α-synuclein transgenic experimental systems exhibiting B-cell reduction as well as in toxin-induced murine PD paradigms, B-cell insufficiency or ablation precipitates exacerbated pathological and behavioral manifestations, substantiating the notion that B cells confer early-stage protection against dopaminergic neuronal degeneration [47]. In the present study, activated B cells were significantly associated only with *B4GALT3*, suggesting that this gene may affect their immune function by regulating modifications in B-cell glycosylation. Beyond B-cell populations, functional aberrations in CD4 T cells demonstrate robust associations with PD pathological features. An altered CD4 T cell migration potential in PD patients, as well as impaired intracellular mitochondrial localization and reduced mitochondrial function, accelerate the progression of the disease. PD-associated transcriptomic signatures, for example, oxidative stress and mitophagy, was found in CD4 + memory T cells suggesting their association with disease pathogenesis [48]. Enhanced α-synuclein expression within murine midbrain regions leads to elevated major histocompatibility complex class II (MHCII) molecule levels in myeloid cell populations of the central nervous system, thereby facilitating the infiltration of IFN-γ-secreting CD4 ⁺ /CD8 ⁺ T lymphocyte subsets [49]. In conclusion, the key genes identified in this study (*B4GALT3*, *GABARAP*, and *RFFL*) may participate in PD pathology by regulating the adaptive immune response, a finding that warrants further investigation for validation.

### 4.4. Regulatory mechanisms of key genes and compound prediction

N6-methyladenosine (m^6^A) is a dynamically reversible eukaryotic RNA modification that regulates the degradation, stability, maturation, and translation of mRNA. When dysregulated, m^6^A can impair dopamine metabolism and affect the function of dopaminergic neurons [50,51]. Studies have shown that an abnormal m^6^A may be an emerging pathological mechanism [50,51]. In this context, the m^6^A methylation sites of key genes were predicted using the SRAMP online tool, and multiple potential modification sites with high confidence were discovered. SRAMP is currently one of the most widely used and highest performing tool for m^6^A site prediction in this field. It should be pointed out that the above prediction results are entirely based on computational biology methods, and there may be inconsistencies between the m^6^A loci predicted via ML and real experimental data; therefore, predictions still need to be rigorously verified by wet experiments in the future. This study reveals for the first time that B4GALT3 and RFFL may influence PD progression through m^6^A-mediated post-transcriptional regulatory mechanisms. Additionally, chromosome 17 (a gene enrichment region associated with neurodegenerative disease) harbors the closely localized *GABARAP* and *RFFL* genes, which may co-regulate protein degradation systems (e.g., autophagy-lysosomal, ubiquitin-proteasome pathways) to influence α-synuclein (α-syn) aggregation [52], providing new evidence for the role of synergistic regulation of key gene clusters in PD genesis. Compound prediction showed that valproic acid and cadmium target *GABARAP* and *RFFL*, whereas phenobarbital acts on RFFL/B4GALT3. Valproic acid (a psychotropic drug for neurological/psychiatric disorders) [53] may offer a new option to treat PD characterized by psychiatric symptoms via GABARAP/RFFL regulation. Cadmium (a toxic metal [54]) impairs synaptic transmission and exacerbates neuroinflammation, implying environmental toxin-related PD mechanisms. Phenobarbital (an antiepileptic drug linked to iatrogenic movement disorders [55]) may aggravate dopaminergic damage by interfering with B4GALT3-mediated glycosylation and RFFL ubiquitination. It is worth noting that recent research on the neuroprotective effects of natural products provides a new perspective with regard to the regulation of the key genes identified in this study. Medicinal and food homologous products derived from Leguminosae species are rich in a variety of bioactive components, which can exert a protective effect on the nervous system through antioxidant and anti-inflammatory properties and pathways that regulate protein homeostasis [56]. In addition, a comparative study found that goji berry and ashwagandha extracts significantly promoted neural stem cell proliferation and reduced neuronal death [57], suggesting that these natural plant extracts can activate neuroprotective signaling pathways and exert anti-PD effects. These findings provide a new intervention strategy for the potential therapeutic targets discovered in this study; that is, compounds derived from natural products may affect PD pathology via epiregulatory or protein-modifying pathways. However, the mechanisms through which these compounds may interact with key genes still need to be experimentally validated.

In this study, by integrating GEO-derived peripheral blood transcriptomic data from PD patients and healthy controls, we identified *B4GALT3*, *GABARAP*, and *RFFL* as key PTMGs involved in PD and subsequently elucidated their functions through various analyses, providing a theoretical basis for the exploration of potential diagnostic biomarkers for PD. However, this study has several limitations. First, the sample sizes of the training and validation sets were relatively small, which may affect the stability and generalizability of the results. Second, peripheral blood transcriptomic data cannot fully reflect pathological changes in the brain. Finally, the screened key genes (*B4GALT3*, *GABARAP*, and *RFFL*) and their regulatory mechanisms still lack validation through in vitro or in vivo experiments. Future studies should, on the one hand, include larger-scale, multicenter independent sample cohorts to perform external validation and repeated analyses of the ML-based prediction model, ROC diagnostic values, and immune infiltration findings, thereby further improving the robustness, reliability, and clinical applicability of the results. However, future research should expand the sample size and include patients of different ethnicities and sexes at different PD stages and conduct multicenter cohort studies to improve representativeness. At the same time, cellular or animal models testing key gene overexpression or knockdown should be constructed to further validate the roles of these genes in PD pathology, including their effects on key events such as neuronal survival, α-synuclein aggregation, autophagy, and neuroinflammation. MeRIP-qPCR or site-directed mutagenesis could be used to further examine the predicted high-confidence m⁶A methylation sites by verifying the molecular mechanisms through which they regulate key gene expression. The screened candidate compounds, such as valproic acid and phenobarbital, should be further tested in vivo and in vitro via pharmacodynamic experiments to elucidate the specific pathways through which they intervene in PD progression by targeting key genes. Advancing these experimental directions will help elucidate the functional network of PTMGs in PD and provide solid experimental evidence for their potential use as early diagnostic biomarkers and therapeutic targets.

## Supporting information

S1 Table Information for 822 posttranslational modification-related genes (PTMGs). (XLS)

S2 Table Pathways identified via GO enrichment analysis of candidate genes. (XLS)

S3 Table Pathways identified via KEGG enrichment analysis of candidate genes. (XLS)

S4 Table Pathways identified via GSEA of the key gene *B4GALT3.* (XLS)

S5 Table Pathways identified via GSEAof the key gene *GABARAP.* (XLS)

S6 Table Pathways identified via GSEA of the key gene *RFFL.* (XLS)
